# Dual Adeno-Associated Virus 9 with Codon-Optimized DYSF Gene Promotes In Vivo Muscle Regeneration and May Decrease Inflammatory Response in Limb Girdle Muscular Dystrophy Type R2

**DOI:** 10.3390/ijms241713551

**Published:** 2023-08-31

**Authors:** Ivan A. Yakovlev, Aleksei M. Emelin, Yana S. Slesarenko, Igor S. Limaev, Iuliia A. Vetrova, Liliya D. Belikova, Ekaterina N. Grafskaia, Pavel A. Bobrovsky, Mikhail V. Pokrovsky, Elena V. Kuzubova, Vladimir M. Pokrovsky, Pyotr A. Lebedev, Sergei N. Bardakov, Artur A. Isaev, Roman V. Deev

**Affiliations:** 1 Genotarget LLC, Skolkovo Innovation Center, 121205 Moscow, Russia; 2 PJSC Human Stem Cells Institute, 129110 Moscow, Russia; 3 Department of Pathological Anatomy, I. I. Mechnikov North-West State Medical University, Ministry of Health of the Russian Federation, 191036 St. Petersburg, Russia; 4 Lopukhin Federal Research and Clinical Center of Physical-Chemical Medicine of Federal Medical Biological Agency, 119435 Moscow, Russia; 5 Faculty of Bioengineering and Bioinformatics, Lomonosov Moscow State University, 119992 Moscow, Russia; 6 Laboratory for Modeling and Gene Therapy of Human Diseases, Belgorod State National Research University, 308015 Belgorod, Russia

**Keywords:** LGMD R2, dysferlin, gene transfer, dual AAV vector, immune response, muscle regeneration

## Abstract

Dysferlinopathy treatment is an active area of investigation. Gene therapy is one potential approach. We studied muscle regeneration and inflammatory response after injection of an AAV-9 with a codon-optimized DYSF gene. A dual-vector system AAV.DYSF.OVERLAP with overlapping DYSF cDNA sequences was generated. Two AAV vectors were separately assembled by a standard triple-transfection protocol from plasmids carrying parts of the DYSF gene. Artificial myoblasts from dysferlin-deficient fibroblasts were obtained by MyoD overexpression. RT-PCR and Western blot were used for RNA and protein detection in vitro. A dysferlinopathy murine model (Bla/J) was used for in vivo studies. Histological assay, morphometry, and IHC were used for the muscle tissue analysis. Dysferlin was detected in vitro and in vivo at subphysiological levels. RT-PCR and Western Blot detected dysferlin mRNA and protein in AAV.DYSF.OVERLAP-transduced cells, and mRNA reached a 7-fold elevated level compared to the reference gene (GAPDH). In vivo, the experimental group showed intermediate median values for the proportion of necrotic muscle fibers, muscle fibers with internalized nuclei, and cross-sectional area of muscle fibers compared to the same parameters in the control groups of WT and Bla/J mice, although the differences were not statistically significant. The inverse relationship between the dosage and the severity of inflammatory changes in the muscles may be attributed to the decrease in the number of necrotic fibers. The share of transduced myofibers reached almost 35% in the group with the highest dose. The use of two-vector systems based on AAV is justified in terms of therapeutic efficacy. The expression of dysferlin at a subphysiological level, within a short observation period, is capable of inducing the restoration of muscle tissue structure, reducing inflammatory activity, and mitigating necrotic processes. Further research is needed to provide a more detailed assessment of the impact of the transgene and viral vector on the inflammatory component, including longer observation periods.

## 1. Introduction

Mutations in the *DYSF* gene, which encodes the dysferlin protein, cause autosomal recessive muscle disorders known as dysferlinopathies. There are several forms of dysferlinopathy: limb girdle muscular dystrophy type R2 (LGMDR2), Miyoshi myopathy (MM), and other phenotypes such as distal myopathy. The DYSF gene is made up of 55 exons that cover 150 kilobases of genomic DNA, and it produces a 6.2-kilobase mRNA transcript. The gene codes for a 237-kilodalton protein that consists of a long N-terminal cytosolic portion that has seven C2 domains, two specific central ferlin domains, and a transmembrane domain at the C-terminus. There is evidence that dysferlin plays a critical role in sarcolemma repair through membrane fusion and vesicle trafficking; dysferlin-deficient myofibers have a reduced ability to reseal membranes after laser-induced damage. A defect in membrane repair plays a role in disease pathology [1,2]. Inflammation has been observed in LGMDR2/MM patients’ muscles; excessive immune response may contribute to the pathophysiology of the disease. The upregulation of inflammasome in dysferlin-deficient muscles suggests that proinflammatory cytokines may be activated in this condition [3]. Muscle biopsies from patients with idiopathic inflammatory myopathies have also shown the presence of cells expressing proinflammatory cytokines [4,5]. Proinflammatory cytokines can trigger the migration of leukocytes, including macrophages, into the muscle tissue [6].

Dysferlinopathy treatment is an active area of investigation. Gene therapy is one potential approach. However, the size of the dysferlin cDNA exceeds the encapsidation capacity of the adeno-associated virus (AAV) vector, which is the most effective and safe vector for muscle identified to date. To overcome this limitation, researchers have employed a strategy with two independent AAV vectors with an overlapping sequence, one of which carries the 5′ end of the cDNA, and the other carries the 3′ end. Injecting both vectors into dysferlin-deficient murine muscles led to the expression of full-length dysferlin, demonstrating that this approach may be useful for gene therapy in LGMDR2 patients.

**Figure 1:**
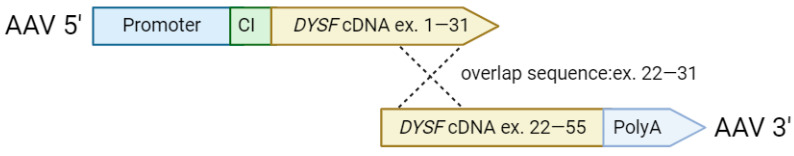
A schematic image of a dual-vector genetic construct AAV.DYSF.OVERLAP. Each part is packaged in a separate virus. AAV5′ contains a part of a dysf cDNA, exons 1 to 31. AAV3′ contains a part of a dysf cDNA, exons 22 to 55. Overlap between two rAAV genomes reconstitutes a split transgene by homologous recombination. CI—chimeric intron.

In this work, we generated an AAV-based dual-vector system (AAV.DYSF.OVERLAP) with a dysferlin cDNA divided into two parts with an overlap sequence (Figure 1) under the control of a muscle-specific promoter. Artificial myoblasts derived from dysferlin-deficient fibroblasts via MyoD overexpression were transduced to evaluate dysferlin mRNA and protein expression. To assess in vivo transduction efficiency and histological changes in muscle fibers, AAV.DYSF.OVERLAP was intramuscularly administrated into transgenic mice B6.A-Dysf^prmd^/GeneJ (Bla/J).

## 2. Results

### 2.1. RT-PCR and Western Blot

**Figure 2:**
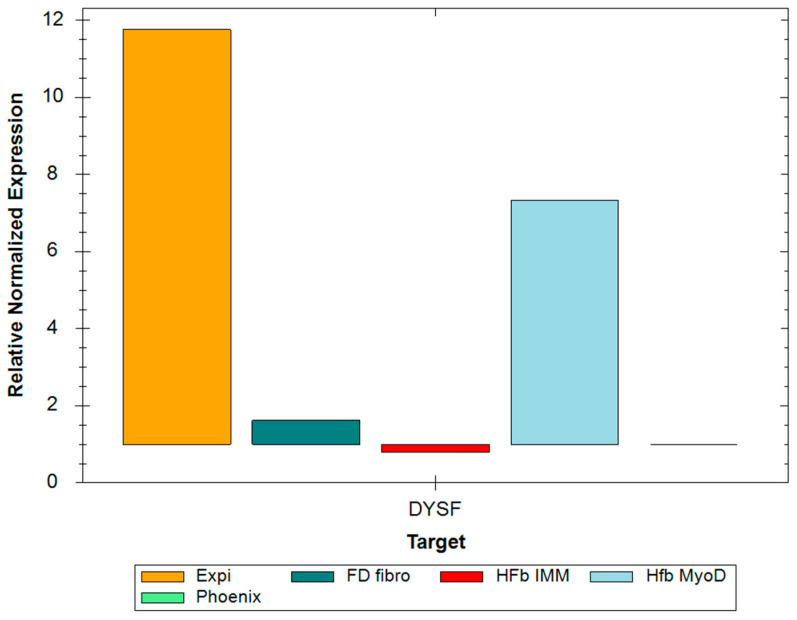
Histogram of the relative change in DYSF gene expression levels. The data are normalized relative to the expression level of the GAPDH gene. The histogram represents the average change in expression level. Expi—Expi293 cells transfected with pCMV-DYSF plasmid; FD fibro—fibroblasts transduced with AAV.DYSF.OVERLAP; HFb IMM—hFb-ex26mut cells transduced with AAV.DYSF.OVERLAP; Hfb MyoD—artificial myoblasts transduced with AAV.DYSF.OVERLAP; Phoenix—Phoenix cells.

For the Expi293 cells transfected with pCMV-DYSF plasmids, as well as HFb mut ex 26 DYSF LV MyoD DOX AAV 72 h cells, an increase in the expression level was observed (Figure 2).

As a result, the presence of dysferlin (expected band at 237 kDa, detected band at 280 kDa according to the antibody manufacturer) was observed in Expi293 cells transfected with the pCMV-DYSF plasmid, as well as in HFb mut ex 26 DYSF LV MyoD DOX AAV 72 h cells.

### 2.2. Histological Assay, Morphometry, and IHC

**Figure 3:**
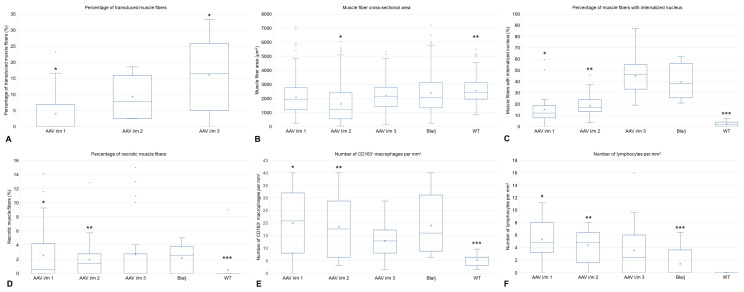
(**A**) Increased transduction efficiency with increasing virus dose. * The presence of statistical significance between the level of transduction of the AAV i/m 1 and AAV i/m 3 groups (*p* = 0.000018). (**B**) Reduced cross-sectional area of muscle fibers in the AAV i/m 2 group compared to other groups, and reduced cross-sectional area in all groups compared to WT. The presence of statistical significance * between the AAV i/m 2 and Bla/J, WT, AAV i/m 1, AAV i/m 3 groups (*p* = 0.001) and ** between the WT and Bla/J, AAV i/m 1, AAV i/m 2 groups (*p* = 0.001). (**C**) Elevated level of muscle fibers with internalized nuclei in Bla/J mouse groups with/without therapy compared to WT, with an increasing trend with increasing virus dose. The presence of statistical significance * between the AAV i/m 1 and Bla/J, AAV i/m 3 groups (*p* = 0.001), ** between the AAV i/m 2 and Bla/J, AAV i/m 3 groups (*p* = 0.038), and *** between the WT and all other groups (*p* = 0.005). (**D**) Decreased percentage of necrotic fibers with increasing administered virus dose, reaching no statistically significant differences between the AAV i/m 3 group and WT. The presence of statistical significance * between the AAV i/m 1 and WT group (*p* = 0.035), ** between the AAV i/m 2 and WT groups (*p* = 0.012), and *** between WT and Bla/j group (*p* = 0.027). (**E**) Decreased inflammatory macrophage (CD163+) infiltration with increasing administered virus dose, reaching no statistically significant difference between the AAV i/m 3 group and WT. The presence of statistical significance * between the AAV i/m 1 and WT group (*p* = 0.001), ** between the AAV i/m 2 and WT groups (*p* = 0.006), and *** between WT and Bla/j group (*p* = 0.022). (**F**) Comparable levels of lymphocytic infiltration in positive and negative control groups, with a decreasing trend in the number of lymphocytes in muscle tissue with increasing virus dose. The presence of statistical significance * between the AAV i/m 1 and WT group (*p* = 0.001), ** between the AAV i/m 2 and WT groups (*p* = 0.003), and *** between Bla/j and AAV i/m 1 group (*p* = 0.036).

**Figure 4:**
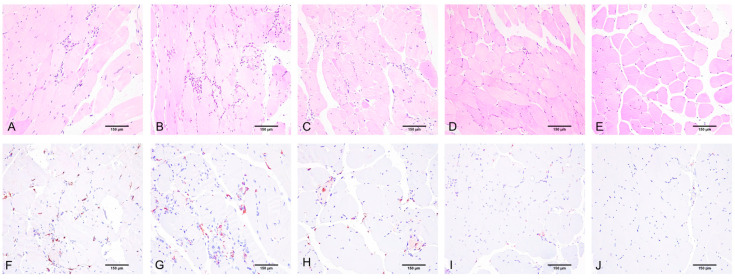
Assessment of structural changes and immune–inflammatory cellular infiltration in skeletal muscles of different mouse groups: (**A**,**F**) Bla/J without therapy; (**B**,**G**) AAV i/m 1 group Bla/j mice with low virus dosage injection; (**C**,**H**); AAV i/m 2 group Bla/J mice with medium virus dosage injection; (**D**,**I**) AAV i/m 3 group Bla/J mice with maximum virus dosage injection; (**E**,**J**) WT. Tendency towards reduction in inflammatory changes in skeletal muscles and restoration to wild-type level (from left to right). Staining: (**A**–**E**) hematoxylin and eosin for evaluating the cross-sectional area of muscle fibers, the percentage of fibers with internalized nuclei, percentage of necrotic muscle, and the number of lymphocytes per mm^2^; (**F**–**J**) immunohistochemical reaction with antibodies against CD163. Counterstained with hematoxylin. Magnification: ×200.

**Figure 5:**
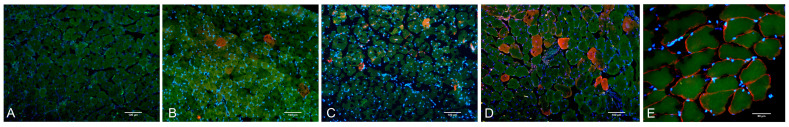
Transverse section of skeletal muscles: (**A**) Bla/J mice without virus injection; (**B**) AAV i/m 1 group Bla/j mice with low virus dosage injection; (**C**) AAV i/m 2 group Bla/J mice with medium virus dosage injection; (**D**) AAV i/m 3 group Bla/J mice with maximum virus dosage injection; (**E**) wild-type (WT) mice. Blue fluorescence spectrum—DAPI; green fluorescence spectrum—autofluorescence of muscle fiber cytoskeleton components; red fluorescence spectrum—result of immunofluorescent reaction with antibodies against dysferlin. Magnification: (**A**–**D**) ×100; (**E**) ×400.

In the AAV i/m 1 group (low virus dosage intramuscularly injection, 5E × 10^11^), the fiber area did not change compared to Bla/J (Figure 3B). The percentage of fibers with internalized nuclei was lower in the AAV 1 group than in Bla/J (Figure 3C). The percentage of necrotic muscle fibers fell in an intermediate position between WT and Bla/J, and was statistically indistinguishable from them (Figure 3D). The extent of macrophage and lymphocyte infiltration in this group is comparable to the Bla/J group, and statistically significantly different from the WT group (Figure 3E,F and Figure 4B,G). The percentage of transduced muscle fibers was predominantly below 15% (median 0%; Q1 = 0%, Q3 = 6.84%) (Figure 5B).

In the AAV i/m 2 group (medium virus dosage intramuscularly injection, 1E × 10^12^), the percentage of necrosis is comparable to Bla/J (Figure 3D). The median fiber area is 40% lower than in Bla/J, which can be explained by the predominance of small muscle fibers, which also accounts for the low percentage of fibers with internalized nuclei (Figure 3B,C). The level of inflammation in this group is comparable to the Bla/J group and is statistically significantly different from the WT group, while the level of lymphocytic infiltration exceeds that of the Bla/J group. The extent of macrophage and lymphocyte infiltration in this group is comparable to the Bla/J group and is statistically significantly different from the WT group (Figure 3E,F and Figure 4C,H). The percentage of transduced muscle fibers exceeded 15% (median 7.69%; Q1 = 2.5%, Q3 = 15.9%) (Figure 5C).

In the AAV i/m 3 group (high virus dosage intramuscularly injection, 5E × 10^12^), the median fiber areas occupy an intermediate position between WT and Bla/J, but they are statistically indistinguishable from the Bla/J group and significantly different from the WT group (Figure 3B). They have a low median level of necrosis, falling in an intermediate position between WT and Bla/J, and are not statistically different from them (Figure 3D). The percentage of fibers with internalized nuclei is high, at the level of Bla/J, which can be explained by the preservation of large muscle fibers (Figure 3C). The activity of infiltration by macrophages and lymphocytes in this group is not statistically different from that in the WT group (Figure 3E,F and Figure 4D,I). The percentage of transduced muscle fibers almost reached 35% (median 16.45%; Q1 = 5%, Q3 = 28.6%) (Figure 5D).

## 3. Discussion

Delivery of the functional DYSF gene into muscle fibers is a complex task because the size of the coding sequence of the DYSF gene exceeds the capacity of AAV vector capsids. Various methods have been proposed to overcome this limitation. Llanga et al. proposed a shortened version of dysferlin called nanodysferlin. Nanodysferlin lacks C2D, CDE, and C2F domains, reducing the cDNA size to 4356 nt. Although the use of AAV–Nano-Dysferlin showed immunofluorescence in approximately 30% of treated fibers [7], there are currently insufficient data on the application of nanodysferlin for muscle tissue restoration in dysferlinopathies. The two-vector system approach has been extensively studied. The dysferlin gene is divided into two parts, with or without the incorporation of additional elements, for intracellular reconstitution of the full-length genetic construct. Several variants have been proposed, including concatemerization–splicing, concatemerization–splicing plus overlapping vectors, and overlapping and fragmented rAAV preparation. The overlapping technology is the most often applied, and data from the research group of Marina Pryadkina and Isabelle Richard suggest that this approach is the most effective [8].

In our study, we used a two-vector overlapping system in which the length of the overlapping sequence was 1387 base pairs. The transgene was packaged in AAV9 and administered intramuscularly at three different doses. The transduction efficiency reached 30% after intramuscular delivery of the viral vector at the maximum dosage. According to the available data, this level of transduction may be sufficient to achieve a clinical effect in the treatment of dysferlinopathies [9,10,11].

In vitro experiments on artificial dysferlin-deficient myoblasts detected transgenic mRNA and dysferlin protein. In vivo, the experimental group showed intermediate median values for the proportion of necrotic muscle fibers, muscle fibers with internalized nuclei, and cross-sectional area of muscle fibers compared to the same parameters in the control groups of WT and Bla/J mice, although differences were not statistically significant. This could be explained by the relatively short duration of 14 days between the administration of genetic vectors and animal slaughter. The inverse relationship between the dosage and the severity of inflammatory changes in the muscles may be attributed to the decrease in the number of necrotic fibers.

An increase in inflammatory infiltration was observed in Group 1, followed by a trend of decreasing inflammatory infiltration with an increase in the dose of the virus. This could be associated with a reduction in necrosis, in groups with higher dosages of the viral vector, which exacerbates inflammation in muscle tissue. The quantity of CD163 macrophages was evaluated. CD163 plays a role in the regulation of inflammation in dysferlinopathy. It is considered a marker of anti-inflammatory macrophages, commonly referred to as M2 macrophages. These M2 macrophages help dampen the immune response and promote tissue repair and regeneration. Studies have shown that dysferlin-deficient muscles exhibit an altered macrophage phenotype, with an increase in pro-inflammatory M1 macrophages and a decrease in anti-inflammatory M2 macrophages. This imbalance can contribute to chronic inflammation and hinder the muscle’s regenerative capacity. A decrease in the level of macrophages in experimental animals could also affect the reactivity to foreign proteins and the AAV capsid itself [12,13,14].

**Figure 6:**
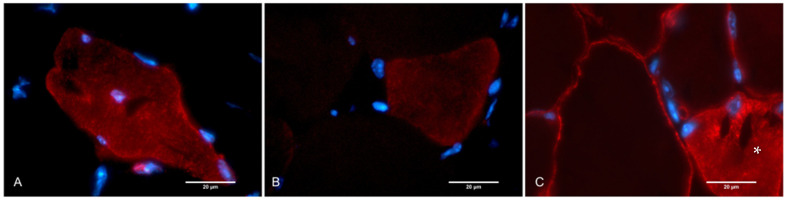
Transverse section of skeletal muscles: (**A**) AAV i/m 3 group Bla/J mice with maximum virus dosage injection—mixed cytoplasmic and membranous pattern of dysferlin distribution in muscle fiber after intramuscular injection of viral vector in Bla/J mouse; (**B**) AAV i/m 3 group Bla/J mice—predominantly membranous pattern of dysferlin distribution in muscle fiber after intramuscular injection of viral vector in Bla/J mouse; (**C**) WT mice, predominantly showing membranous pattern of dysferlin distribution, but cytoplasmic pattern (*) is also observed. Magnification: ×1000.

Different patterns of dysferlin distribution were observed in the cross-sections of animal muscle tissue that were injected with a viral vector (Figure 6). It is known that dysferlin exhibits distinct localization patterns during muscle fiber development. In the early stages of myotube differentiation, dysferlin fails to localize to the plasma membrane and instead accumulates within an internal reticular network [14,15]. This localization pattern, along with previously obtained data, suggests that dysferlin may play a role in T-tubule biogenesis and vesicular transport mechanisms within muscle cells. Indirect observations have pointed to a function for dysferlin in T-tubule biogenesis [6].

Subcellular membrane fractionation studies have revealed that dysferlin associates with both a T-tubule-enriched intracellular membrane fraction and the sarcolemma. This finding aligns with the observation that dysferlin forms an oligomeric complex with the dihydropyridine receptor (DHPR) and caveolin-3, proteins localized in T-tubules. The co-localization of dysferlin and DHPR further supports their interaction and potential involvement in maintaining the stability of DHPR and the ryanodine receptor in the sarcoplasmic reticulum (SR) [15,16,17,18].

Furthermore, dysferlin-carrying vesicles are specifically targeted to disruption sites in the presence of localized high levels of calcium ions. This targeting mechanism leads to the fusion of dysferlin-containing vesicles with each other and the plasma membrane [19].

Dysferlin staining in dystrophic biopsies reveals positive results regarding regenerating fibers, displaying a granular sarcoplasmic pattern. Variation in dysferlin localization is a common characteristic of muscular dystrophies [20,21].

In summary, dysferlin exhibits dynamic localization patterns during muscle development, indicating its potential roles in T-tubule biogenesis, vesicular transport, membrane fusion, and muscle regeneration.

The obtained data suggest that the achieved subphysiological level of dysferlin may be sufficient to reduce the activity of inflammatory and necrotic processes and improve the histological picture as early as 14 days after viral vector administration.

## 4. Materials and Methods

### 4.1. Construction and Production of Recombinant AAV

To create AAV9.DYSF, a previously described codon-optimized sequence of the DYSF gene [22] was used. Plasmid constructs were generated containing the 5′ and 3′ sections of the DYSF gene. The 5′ cassette included the MHCK7 promoter, a chimeric intron, and the first 3370 bp of the dysferlin cDNA, flanked by inverted terminal repeats. The 3′ cassette contained 3866 bp of dysferlin cDNA and the native 3′ non-translated region, including a poly(A) sequence. The plasmid cassettes were used for the separate assembly of double AAV vectors. The double AAV vector included DNA samples with a 1387 bp overlapping region serving as a substrate for recombination to create the codon-optimized dysferlin cDNA (of the canonical dysferlin isoform O75923-1). The pAAV.5′ and pAAV.3′ plasmids were used to generate two separate adeno-associated viruses of serotype 9 following the standard triple transfection protocol [23]. The quality of the AAV9.DYSF.OVERLAP5′ and AAV9.DYSF.OVERLAP3′ viruses was determined by staining on a denaturing polyacrylamide gel (SDS-PAGE).

### 4.2. Construction and Production of Recombinant Lentivirus

Phoenix cells at 70% confluency were transfected with helper plasmids pMD2G (17% by mass of total DNA), psPAX2 (28% by mass of total DNA), and target plasmids LV-TRE-WT human MyoD-T2A-dsRedExpress2 (Addgene (Watertown, MA, USA) plasmid #60628; http://n2t.net/addgene:60628 accessed on 1 June 2022; RRID:Addgene_60628) (55% by mass of total DNA), using the transfection agent TurboFect (Fermentas, Rockville, MD, USA) in a ratio of 22 μL of transfection reagent to 16 μg of DNA per 10 cm Petri dish. The transfection procedure was performed according to the manufacturer’s protocol. After 24, 48, and 72 h, the supernatant containing viral particles was collected, centrifuged for 5 min at 300× *g*, and filtered through a 0.45 μm membrane filter. The collected viral supernatant was aliquoted into 1 mL portions and frozen at −70 °C. One hour before infection, polybrene was added to the culture medium of Phoenix cells, which were in a 20–80% monolayer state, to a concentration of 10 μg/mL. Then, the cells were infected with the viral supernatant at various dilutions (1:1, 1:5, 1:25, 1:125, 1:625).

### 4.3. Fibroblast Preparation

Gingival fibroblasts from a patient with a mutation in exon 26 of the dysferlin gene (hFb-ex26mut) were isolated from a biopsy using the explantation method according to the standard protocol. The gingival biopsies were obtained during surgery with the patient’s informed consent according to the protocol by V.L. Zorin [7].

The fibroblasts were cultured in plastic culture dishes without additional coating in a CO_2_ incubator at 5% CO_2_, 37 °C, and 80% humidity. The medium was changed every 72 h. Once the cells reached the desired confluency, they were washed once with PBS solution and then passaged using 0.25% trypsin solution. The cells were incubated for 5 min under an inverted light microscope to disrupt intercellular contacts and detach the cells from the culture plastic. After that, they were inactivated with an equivalent volume of inactivation medium composed of DMEM (PanEco, Moscow, Russia) + 50 units/mL penicillin–streptomycin (PanEco, Russia) + 10% FBS (Gibco, Miami, FL, USA). The monolayer cells were collected by rinsing the culture dish with a stream of medium from a pipette, transferred to a 15 mL tube, and centrifuged at 200× *g* for 5 min. The supernatant was removed, and the cells were resuspended in fibroblast culture medium. The necessary amount of the cell suspension was then seeded onto the culture plastic.

For cryopreservation, the cells removed from the culture plastic using trypsin and washed with DMEM medium containing 10% FBS were resuspended in FBS and transferred to a cryotube. Then, an equal volume of FBS-20% DMSO mixture was added dropwise to the suspension, and the cells were frozen at −70 °C. The next day, the frozen cells were transferred to liquid nitrogen for long-term storage.

### 4.4. Myogenic Transdifferentiation

The fibroblasts were seeded onto culture plastic without additional coating in a culture medium to achieve 70% confluency. After 48 h, the infection was performed. One hour before infection, polybrene was added to the culture medium of Phoenix cells to a concentration of 10 μg/mL. Then, the cells were infected with the viral supernatant at a quantity of 5 viral particles per cell. After 24 h, a second infection was performed. After 24 h, the culture medium was replaced with fresh medium. After 48 h post-infection, the culture medium for the cells was changed to a medium containing puromycin at a concentration of 1 μg/mL. Selection was carried out for 5 days, with the medium being changed daily. On the 6th day, the culture medium was changed to a medium without puromycin. After 72 h post-transduction, the fibroblasts were detached from the culture plastic using 0.25% trypsin, and the monolayer cell suspension was washed once with PBS solution and resuspended in 300 μL of DPBS without Ca and Mg ions. The cell suspension was transferred to tubes for flow cytometry and sorted using a Melody flow cytometer-sorter. DsRedexpress2-positive cells were collected, using non-transduced fibroblasts as a control. The sorted cells were collected in tubes containing 1 mL of fibroblast culture medium and seeded onto culture plastic.

### 4.5. Animal Models

The study was conducted on transgenic mice B6.A-Dysf^prmd^/GeneJ Bla/J, which carried a retrotransposon in the 4th intron of the dysferlin gene, disrupting the splicing process and dysferlin synthesis (The Jackson Laboratory, Bar Harbor, ME, USA). It has been shown that ultrastructural changes observed in dysferlin-deficient Bla/J mice are correlated with those shown in patients with dysferlinopathy: sarcolemma disintegration, subsarcolemmal accumulation vacuoles, defects in the sarcoplasmic compartment, and lipid droplets in myofibers [24].

AAV.DYSF.OVERLAP viral particles were administered intramuscularly to three groups of animals (*n* = 4) at doses of 5 × 10^11^ (AAV i/m 1; low virus dosage injection), 1 × 10^12^ (AAV i/m 2, medium virus dosage injection), and 5 × 10^12^ (AAV i/m 3, high virus dosage injection) viral genomes in 30 μL of isotonic solution. Doses were based on previous studies assessing dual AAV with dysferlin [10,25]. Intramuscular injection was used to assure the precise administration of viral particles into muscles. Wild-type mice (WT) and Bla/J line mice were used as positive and negative controls, respectively. The administration was performed in the m. gastrocnemius, tibialis anterior, and vastus lateralis muscles. Samples for histopathological analysis were collected after 14 days. Some muscles were fixed in 10% buffered formalin, processed in paraffin using standard techniques, and sectioned at a thickness of 5 μm. Another portion of the muscles was fixed in liquid-nitrogen-cooled isopentane, and cryosections with a thickness of 6 μm were prepared.

### 4.6. PCR

To extract RNA from cells, the samples placed in ExtractRNA reagent were thoroughly homogenized by pipetting. To 1 mL of ExtractRNA reagent containing lysed cells, 0.2 mL of chloroform was added and vigorously shaken for 15 s. The samples were then incubated for 5 min at room temperature and centrifuged for 15 min at 12,000× *g* at +4 °C. After centrifugation, the aqueous phase containing total RNA was collected. Subsequently, total RNA was precipitated with isopropanol, and the pellet was washed with 75% ethanol, dried, and dissolved in 30 μL of DEPC-treated RNAase-free water (deionized water treated with diethylpyrocarbonate and autoclaved). Each sample was treated with 2 units of DNase I (Thermo Fisher Scientific, Waltham, MA, USA) in the presence of 20 units of Ribolock ribonuclease inhibitor (Thermo Fisher Scientific, USA) for 30 min at 37 °C. The reaction was stopped by adding EDTA to a final concentration of 5 mM, followed by incubation at 65 °C for 10 min. The obtained total RNA was used as a template in the reverse transcription reaction. The concentration of total RNA in the sample was determined using the Qubit fluorometer 4 (Thermo Fisher Scientific, USA). For reverse transcription, the RevertAid RT Reverse Transcription Kit (Thermo Fisher Scientific, USA), hexamers, and deoxynucleoside triphosphates were used. The reverse transcription reaction included 1 μg of total RNA, 100 pmol of hexamer primers, 20 units of ribonuclease inhibitor, and 200 units of reverse transcriptase. The reaction components were mixed according to the manufacturer’s recommendations and incubated for 1 h at 42 °C, followed by enzyme inactivation at 70 °C for 5 min. For “RT-” samples, a similar mixture was prepared, but water treated with DEPC was used instead of reverse transcriptase.

Quantitative PCR was performed on a CFX96 Touch thermal cycler (BioRad, Hercules, CA, USA) in a volume of 20 μL using qPCRmix-HS master mix (Eurogene, Moscow, Russia) and 5 pmol of each primer (Table 1). cDNA obtained during reverse transcription, RT- samples, and water were used as negative controls (NTC—no template control).

The relative change in the expression level of the DYSF gene was calculated based on the results of quantitative real-time PCR. The amount of cDNA corresponding to the transcripts of the target and reference genes was determined based on the difference in the threshold cycle (Ct) of the reaction for each sample. The glyceraldehyde-3-phosphate dehydrogenase (GAPDH) gene was chosen as the reference gene. The ∆∆Ct method was used for quantitative gene expression analysis.

In the first step, data normalization was performed using the averaged Ct values of the reference gene GAPDH, and ∆Ct values were calculated by subtracting the Ct value of the reference gene from the Ct value of the target gene. Then, ∆∆Ct values were calculated by subtracting the ∆Ct value of the control sample from the ∆Ct value of the experimental sample. The averaged ∆Ct value for the target gene among the cDNA samples obtained from non-transfected cells (Expi293) was used as the control sample.

To determine the fold change (*FC*) in the expression level, the following formula was applied:

$$FC=2^{-∆∆Ct}$$

For the analysis of the DYSF gene expression level, the primer pairs DYSF_F/DYSF_R and hGAPDH_F/hGAPDH_R were used.

### 4.7. Western Blot

For protein expression analysis using denaturing gel electrophoresis followed by Western blotting, a 1% SDS solution was added to the cell pellet to achieve a protein fraction of approximately 4000 cells in 1 μL of lysate. The cells were disrupted using an ultrasonic disintegrator for 10 s, heated for 10 min at 95 °C, and then centrifuged for 10 min at 14,000× *g*. The resulting supernatant was used to prepare samples for electrophoresis. For this purpose, 15 μL of the lysate (~60,000 cells) was mixed with a 4× sample loading buffer (Table 2), heated for 5 min at 95 °C, and loaded into the gel.

Electrophoresis was performed using the Laemmli method with 6% stacking and 10% separating polyacrylamide gels. Gel casting, protein separation, and equipment such as a gel-casting stand, glass plates, combs, and Mini-Protean Tetra cell system (Bio-Rad, Hercules, CA, USA) were used. The list of solutions used is presented in Table 3. Protein separation was conducted at a constant current of 30 mA per gel in a Tris-glycine running buffer (25 mM Tris-HCl, 192 mM glycine, 0.1% SDS, pH 8.3). Electrophoresis was stopped once the bromophenol blue dye front reached the end of the gel.

Cells used for Western Blot: wild-type human myoblasts cells as a positive control (AB1190 cells were provided by SRC “Biobank” based on FSBI Research Centre for Medical Genetics, Moscow (Head of SRC “Biobank” Tabakov Vyacheslav)); hFb-ex26mut-dysf cells transduced with AAV.DYSF.OVERLAP as a negative control; artificial myoblasts transduced with AAV.DYSF.OVERLAP—experimental cells; Phoenix cells—negative control.

Next, the protein was transferred from the polyacrylamide gel to a PVDF membrane which had been pre-activated with 96% ethanol. For semi-dry transfer, a “sandwich” was assembled, consisting of three layers of filter paper, the gel, the membrane, and three layers of filter paper. All components of the sandwich were pre-wetted in a buffer containing 25 mM Tris-HCl, 192 mM glycine, 2% SDS, 20% ethanol, pH 8.3. The protein transfer was conducted for one hour at a current of 0.8 mA/cm2 of the membrane using a TE77XP semi-dry transfer system (Serva, Heidelberg, Germany).

**Figure 7:**
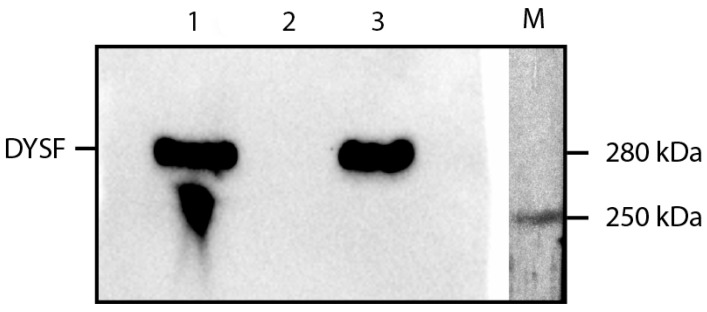
Immunoblot of cell lysates. 1—wild-type human myoblasts; 2—hFb-ex26mut-dysf cells transduced with AAV.DYSF.OVERLAP; 3—artificial myoblasts transduced with AAV.DYSF.OVERLAP; M—molecular weight marker. Staining with antibodies against dysferlin ab124684 (Abcam, Cambridge, UK).

To block nonspecific binding sites on the PVDF membrane, it was incubated with a 2% solution of dry milk (Bio-Rad) in phosphate-buffered saline (PBS) for 40 min at room temperature with shaking at 30 rpm on an MR1 shaker (bioSan, Rīga, Latvia). Subsequently, the membrane was incubated with primary antibodies, specifically ab124684 (Clone: JAI-1-49-3, Uniprot: O75923) (Abcam, Cambridge, UK) diluted 1:1000 for detecting the DYSF protein, overnight at +4 °C. A wash solution containing phosphate-buffered saline with 0.1% Tween 20 was used for washing off unbound primary antibodies. The membrane was washed three times for 10 min each at room temperature with shaking. Following this, the membrane was incubated with secondary anti-rabbit antibodies, specifically NA934 (GE Healthcare, Chicago, IL, USA), for one hour, and then washed three times to remove any unbound antibodies. The antigen–antibody complexes were visualized using the enhanced chemiluminescence (ECL) method with Clarity Max ECL Western Blotting Substrate kit (Bio-Rad, USA) on a Gel Doc XR+ Gel Documentation System (Bio-Rad, USA) (Figure 7).

Cells used for PCR: Expi293 cells transfected with pCMV-DYSF plasmid as a positive control; FD fibro—fibroblasts transduced with AAV.DYSF.OVERLAP as a control for artificial myoblasts; hFb-ex26mut-dysf cells transduced with AAV.DYSF.OVERLAP as an additional control for artificial myoblasts; artificial myoblasts transduced with AAV.DYSF.OVERLAP—experimental cells; Phoenix cells—negative control.

### 4.8. Histological Assay, Morphometry, and IHC

The cryopreserved material, in the form of skeletal muscle tissue samples from Bla/J and WT mice (*n* = 50), was transferred to a Thermo Fisher Scientific HM525 NX cryostat chamber (USA). The samples were oriented on a freezing platform in embedding gel NEG-50 (Richard-Allan Scientific, Dallas, TX, USA) to obtain cross-sectional slices of muscle fibers. After the gel solidified, trimming was performed to a thickness of 100 microns until the slice passed through the entire block. Slices with thicknesses of 3 microns and 7 microns were prepared and transferred onto adhesive-coated glass slides (Epredia, Polysine Adhesion Microscope Slides, Kalamazoo, MI, USA).

The glass slides with cryosections (hereafter referred to as cryosections) of the muscles were dried at room temperature for 20 min. Then, they were immersed in a fixative solution of 10% neutral buffered formalin (Biovitrum, St Petersburg, Russia) for 10 min. After fixation, the cryosections were washed three times for 5 min each in Tris-HCl buffer pH 7.4 (Nevareaktiv, St Petersburg, Russia). Following the washes, a background block solution (Cell Marque, Rocklin, CA, USA) was applied to the cryosections, and they were moved to a humid chamber and incubated for one hour. The protein block was removed by wiping, and then primary recombinant monoclonal rabbit antibodies against dysferlin (Clone: JAI-1-49-3; ab124684; Uniprot: O75923) (Abcam, Cambridge, UK), diluted 1:200 in Diamond antibody diluent solution (Cell Marque, 938B-05, USA), were applied to the cryosections and incubated for 2 h at room temperature in a humid chamber.

After 2 h of incubation, the cryosections were washed three times for 5 min each in Tris-HCl buffer. Subsequently, they were incubated at room temperature in a humid chamber with secondary goat anti-rabbit IgG H&L antibodies (Alexa Fluor 555, ab150078) (Abcam, Cambridge, UK), diluted 1:500 in Diamond antibody diluent solution, for one hour. After one hour, the cryosections were washed four times for 5 min each in Tris-HCl buffer, followed by a 10 min incubation in a working solution of DAPI (Thermo FS, D1306, USA). Then, they were washed twice for 5 min each in distilled water and mounted in a mounting medium—Kaiser’s glycerol gel—with subsequent application of a cover glass.

The histological preparations of skeletal muscle were examined using a fluorescent microscope. Filters were used that allowed the differentiation of light emissions with the following wavelengths: (1) 420 nm (DAPI); (2) 515–530 nm (autofluorescence of muscle fiber cytoskeleton components in the green spectrum for membrane contrast); (3) 565–590 nm (emission of the Alexa Fluor 555 detection system in the red spectrum). Fluorescence of muscle fiber membranes in the red spectrum was considered a positive reaction. Photographs were taken at ×100 magnification, in 10 fields of view for each muscle, using 3 filters, and subsequently overlaid on top of each other. The exposure time used for DAPI was 100 ms, while the exposure times for the Alexa Fluor 555 detection system and green spectrum autofluorescence were both 6.5 s.

The material obtained for histochemical and immunohistochemical analysis, fixed in 10% neutral buffered formalin, was placed in histological cassettes and then washed for 30 min in running water. The cassettes were then processed in a series of alcohols and paraffin according to a manual protocol [23], after which the material was poured into paraffin blocks and microtomed. Microtomy was performed using a Thermo Fisher HM430 rotary microtome, producing 3 μm thick sections. The sections were transferred onto glass slides (Biovitrum, Russia) coated with an adhesive liquid (Biovitrum, Russia). The slides were dried in an oven at 37 °C for 12 h, followed by deparaffinization, rehydration, staining with hematoxylin (Biovitrum, Russia) and eosin (Biovitrum, Russia), dehydration, clearing, and mounting with a mounting medium based on polyester under a cover glass.

For the evaluation of inflammatory macrophage infiltration in skeletal muscles (*n* = 20), an immunohistochemical reaction was performed using monoclonal rabbit antibodies against CD163 [ERP19518] (ab182422, Abcam, UK), diluted in Diamond antibody diluent (Cell Marque, 938B-05, USA) at a ratio of 1:500, with a 2 h incubation at 37 °C in a humid chamber. The detection system used secondary antibodies Goat Anti-Rabbit IgG H&L (HRP) (ab205718, Abcam, UK) with an immunoperoxidase label, diluted to 1:5000, and a 2 h incubation at 37 °C in a humid chamber. The reaction was visualized under a microscope using a DAB Substrate kit (957D-21, Cell Marque, USA), with subsequent counterstaining with Mayer’s hematoxylin solution.

The photodocumentation was performed using a computer video system (IBM PC + Leica DM 1000 microscope, Wetzlar, Germany) and the ImageScope-M software package (https://www.microscop.ru/oborudovanie/drugoe/item/imagescoupe/ accessed on: 1 May 2022) (Moscow, Russia). Photographs were taken at a magnification of ×200, capturing 10 fields of view for each muscle sample. The following parameters were selected for evaluation in the morphometry of hematoxylin–eosin-stained sections: the cross-sectional area of muscle fibers (the cross-sectional area represents the area of one muscle fiber in a section that passes perpendicular to the axis of the muscle fiber), the percentage of fibers with internalized nuclei and necrosis relative to the total number of fibers in the field of view, and the number of lymphocytes per mm^2^. For the assessment of inflammatory macrophage infiltrate in the muscles, the number of CD163^+^ macrophages per mm^2^ was used as an indicator.

When working with immunofluorescence microscopy data, the efficiency of transduction was evaluated using an index formed by the ratio of the number of muscle fibers with membrane fluorescence in the red spectrum to the total number of muscle fibers in one field of view, multiplied by 100%. Morphometry was performed only on tissue samples with introduced AAV viral.

Statistical analysis was conducted using the IBM SPSS Statistics 26 software (Armonk, NY, USA). To determine the statistical significance of differences between groups, the non-parametric Kruskal–Wallis rank test was used, with Bonferroni correction for multiple comparisons.

## 5. Conclusions

The use of two-vector systems based on AAV is justified in terms of therapeutic efficacy. The expression of dysferlin at a subphysiological level, within a short observation period, is capable of inducing the restoration of muscle tissue structure, reducing inflammatory activity, and mitigating necrotic processes. Further research is needed to provide a more detailed assessment of the impact of the transgene and viral vector on the inflammatory component, including longer observation periods.

## Figures and Tables

**Table 1:** List of oligonucleotides used in the study.

Primer	Sequence
hGAPDH_F	TCGGAGTCAACGGATTTGGT
hGAPDH_R	TTCCCGTTCTCAGCCTTGAC
DYSF_F	CGGTGGGCCATCATTCTGTTT
DYSF_R	TCAGGAGAATGGCTTCACCAGTTTCAT

**Table 2:** Composition of 4× sample loading buffer.

Component	Buffer Contents
Tris-HCl, pH 6.8	200 mM
β-mercaptoethanol	400 mM
SDS	4%
bromophenol blue	0.01%
Glycerol	40%

**Table 3:** List of reagents required for casting one separating and one stacking gel.

	Separating Gel	Stacking Gel
Volume	4 mL	2 mL
% Acrylamide (AA)	10%	6%
Water	1.7 mL	1.4 mL
AA:bis-AA (30%)	1.3 mL	0.33 mL
Buffer	1.5 Tris (pH 8.8) 1 mL	1.0 Tris (pH 6.8), 0.25 mL
SDS 10%	40 μL	20 μL
PSA 10%	40 μL	20 μL
TEMED	6 μL	4 μL

## Data Availability

The data presented in this study are available upon request from the corresponding author. The data are not publicly available due to a non-disclosure agreement.
